# ﻿Review of the genus *Prochasma* Warren (Geometridae, Ennominae, Boarmiini), with description of a new species from Hainan, South China

**DOI:** 10.3897/zookeys.1190.112468

**Published:** 2024-01-31

**Authors:** Bo Liu, Dieter Stüning

**Affiliations:** 1 Coconut Research Institute, Chinese Academy of Tropical Agricultural Sciences, Wenchang, 571339, China Coconut Research Institute, Chinese Academy of Tropical Agricultural Sciences Wenchang China; 2 Leibniz Institute for the Analysis of Biodiversity Change-Zoological Research Museum Alexander Koenig, Adenauerallee 124, D-53113, Bonn, Germany Leibniz Institute for the Analysis of Biodiversity Change - Zoological Research Museum Alexander Koenig Bonn Germany

**Keywords:** Checklist, COI, key, male genitalia, morphology, *P.diaoluoensis* sp. nov., taxonomic history, taxonomy

## Abstract

The few already published generic features of the genus *Prochasma* Warren, 1897 are reviewed and new-found characters are added to make the generic description more comprehensive. A new species, *Prochasmadiaoluoensis* Liu & Stüning, **sp. nov.** is described from Hainan Province, China. It is the only *Prochasma* species found on this island and exceptional for its conspicuous pattern, vivid coloration and some morphological characters not observed in other species before. Descriptions and illustrations of adults, their venation, and male and female genitalia are presented. An identification key and an annotated checklist of all presently known species of *Prochasma* are provided. In addition, a DNA barcode sequence is given for the new species, and preliminary phylogenetic estimations of the genus *Prochasma* are discussed.

## ﻿Introduction

The genus *Prochasma*, now belonging to the tribe Boarmiini in the subfamily Ennominae, was erected by Warren (1897) with *P.mimica* Warren as its type species and Khasi Hills, India as its type-locality. As a comment following the description, Warren admitted that his “*dentilinea*, wrongly referred to *Psilalcis*” (Warren 1893: 431) is extremely similar to *Prochasma*, but also mentioned differences in neuration and wing pattern, so he did not formally transfer *dentilinea* to *Prochasma*. Hampson (1895) added *Psilalcisdentilinea* to his large concept of *Boarmia* (*Psilalcis* was, like many other genera of Boarmiini, synonymized with *Boarmia* before). Later Hampson (1898: 724) erroneously proposed to add “var. pulverosa Warren”, which was described as “EctropisdentilineataMooreab.pulverosa nov.” by Warren (1896: 403), to *dentilinea* Warren. On the same page he provided the (unnecessary) replacement name *Boarmiaflavisecta* Hampson, 1898, nomen novum, for *Prochasmamimica*, which he wrongly cited as *P.* “*minima*” and had found to be preoccupied in his genus *Boarmia*. Almost 30 years later, *Psilalcisdentilinea* Warren was transferred to *Prochasma* by Prout (1926) mainly based on the presence of the metallic mesothoracic crest, and he also described two more species: *P.scissivestis* Prout, 1926 from Sarawak, Borneo, and *P.albimonilis* Prout, 1927 from Htawgaw, NE Burma. In the latter paper, Prout also questioned the nomenclatoric treatment of Hampson (1898) (see above), but did not correct it. Parsons et al. (1999) listed the four abovementioned species as members of the genus *Prochasma*, but added a further name, *P.squalida* Wileman, 1915, described as “*Boarmia*” from Taiwan, as a synonym of *P.dentilinea*. Sato (2019) revised *Prochasma* and described two new species, *P.kishidana* from Peninsular Malaysia, Sumatra and Borneo, and *P.sasakiana* from Borneo only, as well as restoring *P.squalida* as a distinct species, and transferring *P.scissivestis* Prout, clearly misplaced in *Prochasma*, correctly to the genus *Alcis* Curtis, 1826, as a member of the “*pammicra*-complex” (Sato 2005). Rajaei et al. (2022) listed six species-names in *Prochasma*, with *P.dentilinea* incorrectly as a junior synonym of *P.squalida*. A further new species was later described as *P.parasqualida* by Sato (2023), based on specimens from Vietnam, Laos and Thailand. These specimens were earlier treated as conspecific with *P.squalida* from Taiwan, because of their distinctive similarity of pattern and genitalia. Up to now, there are seven species recorded in the genus *Prochasma*.

Recently, many specimens of *Prochasma* have been collected on Hainan Island, China, which could be confirmed as new to science and will be described here.

## ﻿Materials and methods

### ﻿Materials

All specimens of the new species were collected by light traps on Hainan Island, S. China and currently are deposited in Coconut Research Institute, Chinese Academy of Tropical Agricultural Sciences, Wengchang, China (**CRICATAS**). For long-term preservation, most of the type specimens of the new species, including the holotype, will be transferred to the Institute of Zoology, Chinese Academy of Sciences, Beijing, China (**IZCAS**) and some of the paratypes will be transferred to the Zoologisches Forschungsmuseum Alexander Koenig, Bonn, Germany (**ZFMK**).

### ﻿Morphology

Terminology for wing venation followed the Comstock-Needham System (Comstock 1918) as adopted for Geometridae by Scoble (1992) and Hausmann (2001), and that of the genitalia was based on Klots (1970) and Skou and Sihvonen (2015). For genitalia examination, abdomens were removed and placed in 10% NaOH solution. Genitalia were dissected in purified water and stained with Chlorazol Black E. Photographs of adults were taken with a Nikon camera (model: D750) equipped with a Nikon lens (AF-S Micro 60 mm f/2.8G ED). Photos of genitalia were taken with a digital camera (KUY NICE E31SPM) attached to a Nikon microscope (model: SMZ745T). Focus stacking images (20 to 30 stacks in 0.25 mm increments were used for each adult image) were generated using Helicon Focus (version: 8.2.2 pro) software.

### ﻿DNA barcoding

Genomic DNA was extracted from the legs of dried adult specimens and the barcode fragments were amplified using primers pairs: LCO-1490 and HCO-2198 (Folmer et al. 1994). The PCR products were recovered and cloned and the positive plasmids were sequenced by Sangon Biotech Co., Ltd (Shanghai, China). The obtained sequence information was deposited in the Barcode of Life Data Systems (BOLD: Ratnasingham and Hebert 2007). All sequences utilized in this study, with the exception of the newly described species, were obtained from BOLD Systems. Sequence divergence within and between species was calculated using the Kimura 2-parameter model (Kimura 1980) and the neighbour-joining algorithm (Saitou and Nei 1987), as implemented in BOLD Systems. Genetic distances within and between species are reported as uncorrected pairwise distances (p-distance). Phylogenetic tree construction and species divergence calculations were performed using MEGA 11 (Tamura et al. 2021).

## ﻿Taxonomic account

### ﻿ Prochasma

Taxon classificationAnimaliaLepidopteraGeometridae

Warren, 1897

0EFF45D0-CAB9-51D8-B623-F9FA01FAFD18

Prochasma Warren, 1897, Novit. zool. 4: 81. Type species: Prochasmamimica Warren, 1897.

#### Diagnosis.

The genus *Prochasma* Warren currently comprises a total of eight species, including the newly described species presented in this study. These species are united by an apomorphic character, a tuft of well-developed, basally narrow, distally broad and curved, upright scales with metallic gloss on the posterior part of mesothorax in both sexes. This character is unique and distinguishes *Prochasma* from other genera of the tribe Boarmiini, though curved, light-reflecting scales also occur in a number of other geometrid groups; however, these scales are not arranged as an upright brush and other characters such as antennae, transverse lines, fovea (present), venation, male and female genitalia etc. are quite different. There is no genus comparable in size and pattern known to us, with which *Prochasma* could be confused.

#### Generic description.

A generic description was provided, besides the original description by Warren (1897), only by Holloway [1994] and by Sato (2019); the latter partly repeated the characters mentioned by Holloway, but added some new features. Herein, we summarize these already published characters of *Prochasma*, with a few corrections, and add new-found, unpublished features.

**Figure 1.**
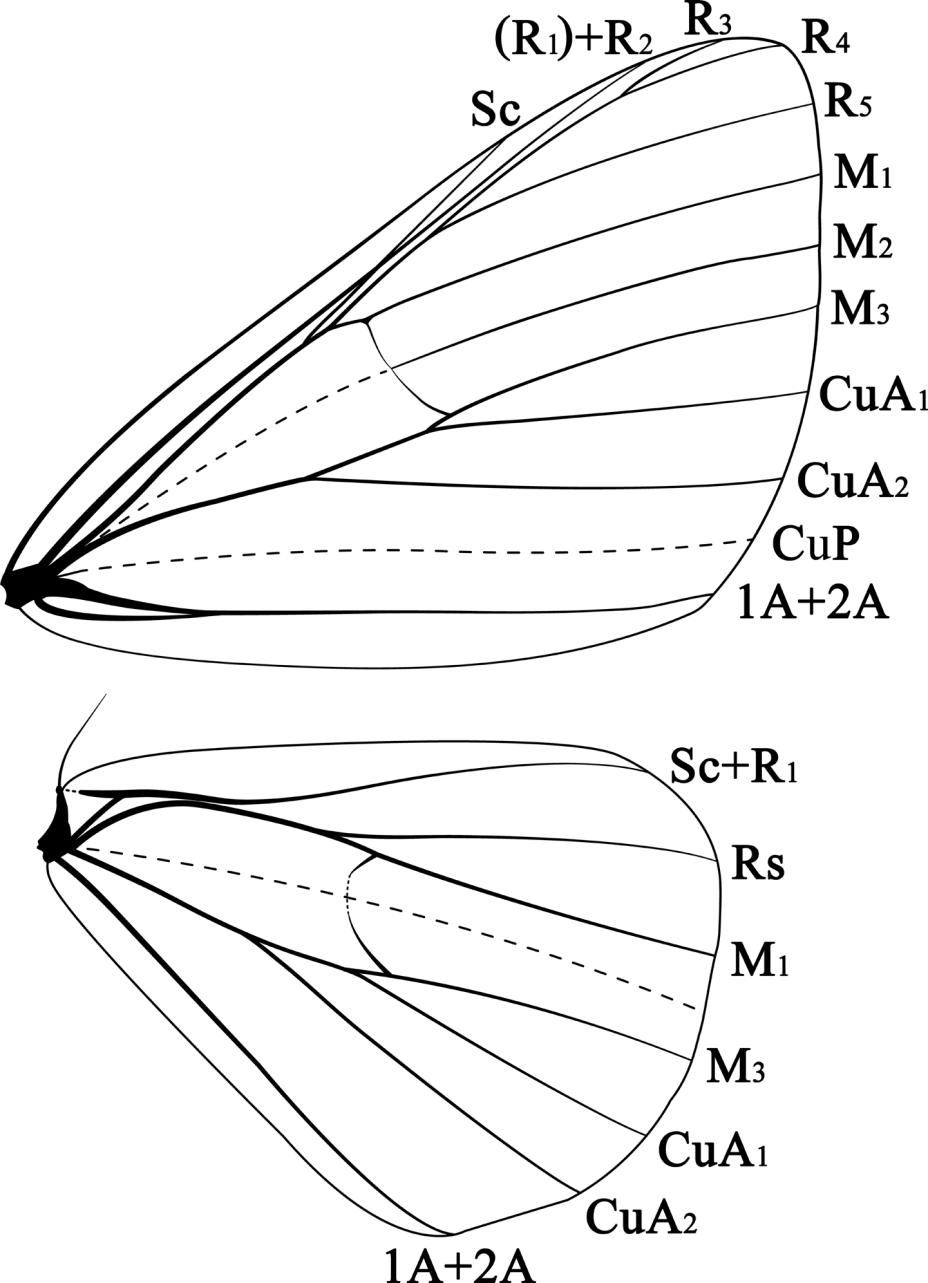
Wing venation of *Prochasmadiaoluoensis* sp. nov.

***General appearance.*** Tiny ennomine moths, wingspan 18–25 mm, forewing length: male 10–15 mm, female 11–16 mm, with colourful, yellow, white and black pattern, medial zone of forewings dark in most species. ***Head*.** Male antennae bipectinate, rami arising from basal end of each segment, dorsally unscaled, densely ciliate ventrally, apical one-third of flagellum non-pectinate; female antennae filiform (not “fasciculate”, as mentioned by Sato 2019: 138). Frons narrow, rather flat, smooth-scaled, palps curved upwards before frons. Proboscis short, but functional. Chaetosemata present, small, near eye-margin. ***Thorax*.** Patagia and tegulae with large, lamellar, partly elongated scales, tegulae in addition with long hair-scales. Mesothorax posteriorly (on mesoscutellum) with a tuft of large, distally curved, metallic scales in both sexes (see Sato (2019, fig. 25); also mentioned by Warren (1897) and Holloway [1994]). Forewing pale yellow or grey (*P.albimonilis*), with distinct dark markings, without fovea in males. Antemedial and postmedial lines thin, black, deeply incurved and outwardly dentate, in some species, reduced to short streaks or dots, bounded distally by a narrow or broader band of the pale ground colour (on proximal side in antemedial lines). Submarginal lines narrow, white, zigzag-shaped where visible. In forewings, the dark band on the outside of the postmedial line broad, variable individually and in different species. The dark band on the inner side of the submarginal line on hindwing also variable in breadth, sometimes narrower, sometimes extending to the costa, angled outside or reduced to a spot. Discal dot distinct or small, black, visible on both wings, but larger on forewings. Underside similar to upperside, but more blurry and paler. Legs slender, light grey, chequered dark grey or black. Index of spurs 0-2-4, hind tibia hardly swollen, with two pairs of long spurs and with a whitish scent brush in males. ***Venation*** (Fig. 1). R_1_ and R_2_ coincident (distal branch of R_1_ reduced, only R_2_ reaching costa), the base of the combined veins running closely parallel to vein Sc or anastomosing with it for a short distance. Other veins inconspicuous, vein 3A in hindwing absent. Folds through cells of both wings and those replacing CuP in forewings and M_2_ in hindwings very vague. ***Pregenital abdomen*.** Tergites and sternites not conspicuously modified. T1 and T2 sclerotized, T1 narrow, T2 of double breadth. Seventh segment distinctly narrow, eighth segment elongate. Coremata absent. Tympanal organs of moderate size, without lacinia. Setal comb present, but modified to a multi-row setal patch, with numerous small, easily detachable setae; when central setae are lost, it may look like “a pair of setal scars” (compare Holloway [1994: 269] and Sato (2019, fig. 24)). Sterno-tympanal process present, but weak, free distal portion short, not reaching the posterior margin of tympanal bulla.

***Male genitalia*.** Uncus triangular, base broad, lateral sides almost straight or slightly rounded, with short setae dorsally, apex short and pointed or more or less narrowly elongated and pointed. Gnathos with strong lateral arms, central part strong, elongate rectangular, with rounded tip. Juxta broad, plate-like, sclerotized, with distal incision in some species or rectangular, with apex slightly narrowed. Saccus strong, triangularly more or less extended, tip rounded. Valvae elongated, parallelogram-shaped in some of the species, sclerotized costa not reaching the weak, narrowed distal part of valvae, which is covered with a moderate to weak cucullus, which is reaching widely basad. Tip of valvae rounded, rarely dorsal margin deeply excavated more basally and carrying a tuft of long, modified setae (so far only found in the new species described below). Ventral margin of valvae at 2/3 to 3/5 length with a short, tooth-like process (i.e., distal process of sacculus). The latter is built as a narrow, sclerotized band along ventral margin of valva and may sometimes be weak or even not visible; distal tooth-like process is variable in size and may rarely be short or almost absent. Basal part of valve lamina less setose and more or less membranous, bordered distally by an oblique, sclerotized ridge. Aedeagus short and stout, vesica containing a single massive cornutus, with significant variations of size and shape between different species, an important specific character.

***Female genitalia*.** Ovipositor short, papillae anales narrow, tapering, covered with short setae. A needle-like sclerite, found between the bases of posterior apophyses in two species so far, may also turn out to be a generic feature. Anterior apophyses distinctly shorter than posterior apophyses, the latter almost double in length. Introitus bursae funnel-shaped, often large, slightly sclerotized. Colliculum absent (Sato (2019: 141) mentions it as “developed”, but we could not confirm the presence of a typical colliculum). Posterior part of bursa copulatrix elongated, largely membranous, posteriorly with various types of specifically different sclerotizations. Anterior part of bursa membranous, slightly broader than posterior part, but no clear demarcation visible. Signum absent.

### ﻿ Prochasma diaoluoensis sp. nov.

Taxon classificationAnimaliaLepidopteraGeometridae

4DC430C2-7F94-52A4-B1DC-3AEB59CF55FB

https://zoobank.org/920827A8-1F55-4604-99B7-6B89898E0366

Figs 2–7 , 8, 9 , 10–14

#### Type-material.

***Holotype***: male, China, Hainan Province, Lingshui, Diaoluoshan, 922 m, 20.IV.2023, Bo Liu leg. DNA barcode CRICATAS00001 (CRICATAS/ IZCAS). ***Paratypes*** (67 males, 7 females): 13 males 3 females, same locality and collector as holotype, 20.IV.2023, gen. prep. no. CRICATAS00064; 39 males 4 females, same locality and collector as holotype, 10.V.2023, gen. prep. no. CRICATAS00063, gen. prep. no. CRICATAS00071; 6 males, same locality and collector as holotype, 19.VI.2023; 9 males, same locality and collector as holotype, 19.VIII.2023. (CRICATAS/ IZCAS/ ZFMK).

**Figures 2–7.**
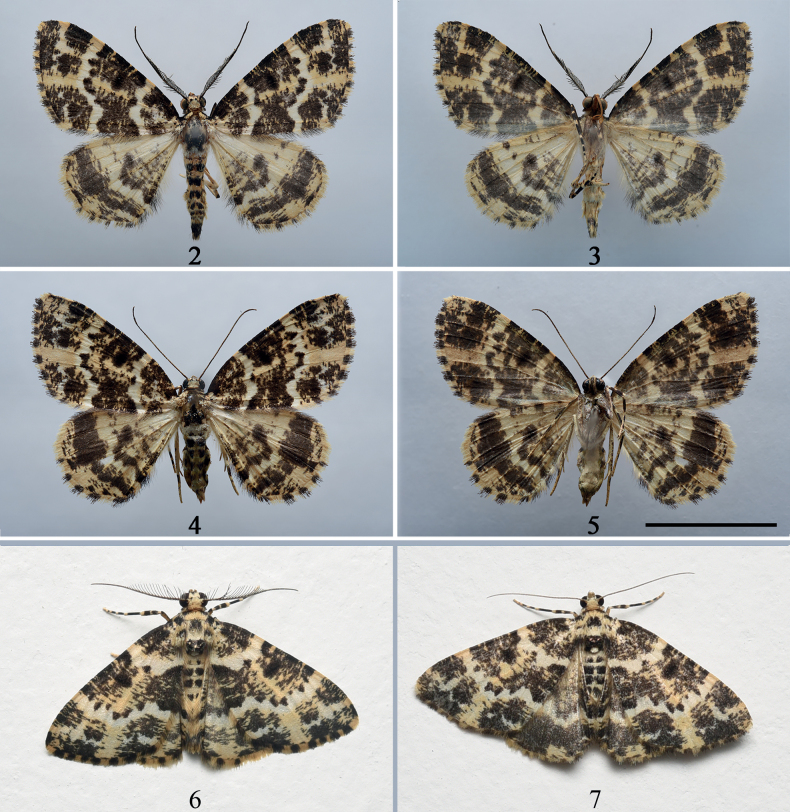
Adults of *Prochasmadiaoluoensis* sp. nov. **2** male, holotype, upperside **3** male, holotype, underside **4** female, paratype, upperside **5** female, paratype, underside **6** male, paratype, living specimen **7** female, paratype, living specimen. Scale bar: 1 cm.

#### Diagnosis.

*Prochasmadiaoluoensis* is distinguished from its congeners by the following characteristics: 1) Valvae with a deep excavation on dorsal side near apex, basally adjacent a brush of modified setae present, absent in other species (Sato 2023, in litt.); 2) Apex of uncus very short, not narrowly elongated; 3) female genitalia with an elongate, funnel-shaped, sclerotized structure on posterior part of bursa copulatrix and a spoon-shaped lamella postvaginalis; and 4) Ante- and postmedial lines reduced to small denticles, bordered by broad, white lines, more conspicuous than in the congeners. The latter two features are found, but less expressed, also in some other species. The same can be stated about the horizontal, yellow band, traversing both forewings, which is most conspicuous in *P.mimica*, less conspicuous in *diaoluoensis*, but often present, at least in traces, also in the other congeners. Generally, the new species is, though more vividly coloured and with more strongly contrasting pattern, rather similar to its congeners, with exception of *P.albimonilis* which lacks the yellowish ground colour and has homogenous, dark grey pattern elements, almost not separated into basal, medial and postmedial areas. The female genitalia of *albimonilis* are similar to *P.diaoluoensis* in the posterior part of bursa, which is also roundly extended on right side, but the sclerotized part is not funnel-shaped but rather broadly tube-like (Sato 2019, fig. 40). It may even be a functional colliculum. In male genitalia, a narrow dorsal incision is present near apex of valvae in *albimonilis*, but the valvae are broader, especially the sclerotized costal side, and more densely setose.

#### Description.

***Forewing length***: male 12.2–13.2 mm; female 12.9–13.6 mm. ***Head*.** Antennae bipectinate on basal two-thirds in males, rami long, length of longest rami about 9 times the diameter of the flagellum segments, filiform in females. Frons not protruding, covered with short scales, upper half pale, lower half dark. Labial palpus curved upwards beyond frons, covered with intermingled, dark and pale scales and longer hair-scales. Vertex with pale scales, a few dark scales near antennae. ***Thorax*.** Patagia and tegulae with lamellar, dark and pale scales, with longer, dark hair-scales on tegulae only, ventrally thorax covered with pale yellow hair-scales. Legs slender, pale, chequered black, hind tibia slightly dilated, with a pale scent brush in males. Forewings with apex angled, termen smoothly curved, without fovea in males. Hindwing with apex rounded. Wings yellow, covered with extensive black scales. Fringes with alternating yellow and smaller black parts. Forewing yellow, with distinct dark markings. Antemedial and postmedial lines both appear as consisting of a few black denticles or dots between M_1_ and CuA_2_, bordered by a broad, white band. In females, the denticles are more tooth-like. Submarginal line white, very fine, zigzag-shaped. Area between M_3_ and CuA_1_ appears as a yellow, horizontal band, with or without a few small black spots. Discal dot oval, black, faintly visible. Dark band on inner side of postmedial line of hindwing narrow, reaching from discal dot to inner margin. Dark band on outside broader, the width variable between individuals, slightly broader in females. Submarginal line visible, intermittent, weaker in hindwings. Underside similar to upperside, but more blurry and paler. ***Venation*** (Fig. 1). Forewing: R_1_ and R_2_ coincident; R_1_+R_2_ arising from upper vein of cell, then shortly anastomosing with Sc, and running almost parallel to the long stem of R_3-4_; stem of R_3-5_ arising shortly before anterior angle of cell; M_2_ from the middle of the discocellulars; CuA_1_ from before posterior angle of cell. Hindwing: Sc+R_1_ running closely parallel but not anastomosing with upper vein of cell at base; Rs from before anterior angle of cell; CuA_1_ from before posterior angle of cell; 3A absent. ***Pregenital abdomen*.** Dorsally scaled pale yellow, with a large black spot on each tergite. Ventrally with pale yellow hair-scales. Tympanal organs and a modified setal comb present, the latter as described in the generic description. Tergite and sternite of segment 7 short, length about 2/5 of width. Tergite and sternite of segment 8 elongate, length slightly greater than width in males, broader in females.

**Figures 8, 9.**
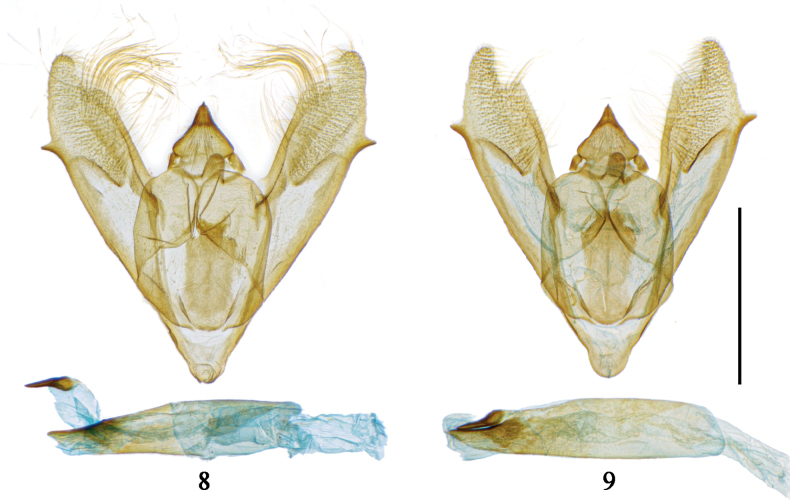
Male genitalia of *Prochasmadiaoluoensis* sp. nov. **8** paratype (vesica partly everted), gen. prep. no. CRICATAS00063 **9** paratype (brushes of modified setae removed, vesica not everted), gen. prep. no. CRICATAS00064. Scale bar: 1 mm.

***Male genitalia*.** Uncus triangular, base broad, with short setae dorsally, apex very short, not narrowly elongated. Gnathos with strong lateral arms, central part strong, rectangular, with rounded tip. Juxta rectangular, sclerotized, apex slightly narrowed. Saccus V-shaped, slightly extended. Valvae elongated, apically narrowed ventrally, tip rounded, with a deep excavation on dorsal side. Valve lamina proximally membranous, distally densely covered with setae, without a typical cucullus, with an oblique, sclerotized ridge between both parts. A tuft of long, curved, modified setae, tubular at base, distally flattened, present dorsally near the apex of each valva. Costa straight, sclerotized, basally slightly broadened, distally not reaching tip of valva, ending at excavation. Sacculus sclerotized, distally with a short, tooth-like process, protruding from ventral margin of valva at ¾ of its length. Aedeagus cylindrical, apically broadly elongated and sclerotized on one side. Cornutus short, not stick-like, apex tapering, with bulbous base.

***Female genitalia*.** Ovipositor short, papillae anales narrow, tapering towards apex, covered with short setae. Anterior apophyses short, about 2/3 length of posterior apophyses. A thin needle-like sclerite, roundly enlarged anteriorly, present between the bases of posterior apophyses. Lamella postvaginalis large, spoon-shaped. Introitus bursae funnel-shaped, slightly sclerotized. Posterior part of bursa elongated, membranous, distally roundly extended on right side; outside with a posteriorly funnel-shaped sclerotized structure formed by a broad sclerite which consists of lamellar plates folded three times, with unknown function (see Figs 11–14). Anterior part of bursa slightly broader than posterior part, but no clear demarcation visible. Signum absent.

**Figures 10–14.**
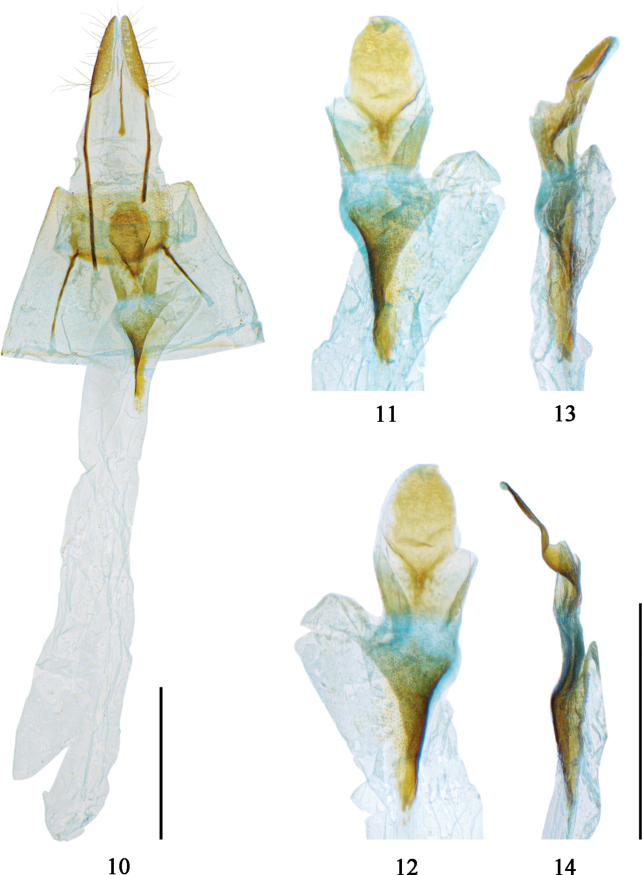
Female genitalia of *Prochasmadiaoluoensis* sp. nov. **10** paratype, gen. prep. no. CRICATAS00071 **11–14** Close-ups of lamella postvaginalis, introitus bursae and posterior part of bursa **11** ventral view **12** dorsal view **13** lateral view from right side **14** lateral view from left side. Scale bars: 1 mm.

#### Etymology.

The specific name is derived from the type-locality, Diaoluoshan, Hainan Island, China.

#### Distribution.

China (Hainan).

##### ﻿Preliminary phylogenetic estimations

**Figure 15.**
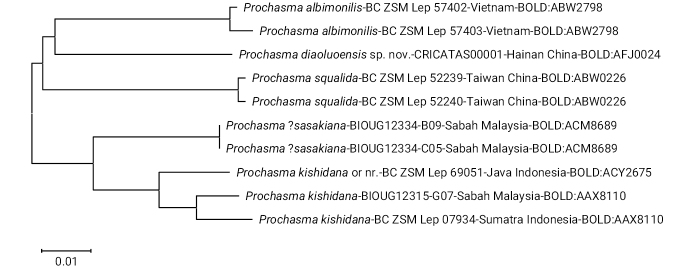
Neighbour-joining tree of *Prochasma* taxa on the basis of DNA barcoding.

A barcode sequence based on the COI (658 bp) was obtained from the holotype of *P.diaoluoensis* and submitted to BOLD Systems (BIN: BOLD: AFJ0024, Sample ID: CRICATAS00001, Process ID: CCLEP001-23). There are currently 14 (including the one for *P.diaoluoensis*) *Prochasma*-associated DNA barcoding records on BOLD Systems. Four of them are private and restricted to use only within BOLD Systems, and the remaining ten published records are available but contain nonidentifications and misidentifications. On the basis of the images of the specimens provided on BOLD, the locality information attached to the records and the provided barcode data, most specimens could be identified to species level. However, three species (*P.mimica*, *P.dentilinea*, *P.parasqualida*) are still not represented on BOLD, so a full phylogenetic analysis of the genus is not yet possible. However, based on the data currently available, the following preliminary conclusions can also be drawn: The neighbour-joining tree of *Prochasma* (Fig. 15) clearly shows that *P.diaoluoensis* is a distinct species and most closely related to *P.albimonilis*, with a mean genetic distance of 7.05% (p-dist) (Table 1). Interspecific genetic distances range from 4.9% to 8.7%, intraspecific values range from 0.3% to 2.0%. Furthermore, a phylogenetic tree, offered and constructed by BOLD Systems and based on the sequences of the “100 nearest neighbours”, i.e., the species most closely related to *P.diaoluoensis*, showed that all sequenced *Prochasma* species clustered into a single clade of the phylogenetic tree. This is consistent with the results of our morphology-based study (see generic description and diagnosis). The three not yet sequenced species (i.e., *P.mimica*, *P.parasqualida*, *P.dentilinea*) largely agree with the morphological characters of the others and will not change the homogenous character of the cluster, then representing the genus *Prochasma*.

**Table 1.** Genetic distances (p-distance) within and between species of the genus *Prochasma*.

		1	2	3	4	5	6	7	8	9	10
1	*P.diaoluoensis*-CRICATAS00001										
2	*P.albimonilis*-BC ZSM Lep 57402	6.8%									
3	*P.albimonilis*-BC ZSM Lep 57403	7.3%	0.6%								
4	*P.squalida*-BC ZSM Lep 52239	7.4%	7.8%	7.8%							
5	*P.squalida*-BC ZSM Lep 52240	7.8%	7.8%	7.4%	0.3%						
6	P.?sasakiana-BIOUG12334-B09	7.3%	7.8%	8.2%	7.6%	7.6%					
7	P.?sasakiana-BIOUG12334-C05	7.3%	7.8%	8.2%	7.6%	7.6%	0.0%				
8	*P.kishidana* or nr.-BC ZSM Lep 69051	7.8%	8.1%	8.2%	7.8%	7.8%	4.9%	4.9%			
9	*P.kishidana*-BIOUG12315-G07	7.6%	7.3%	7.5%	8.2%	8.2%	5.6%	5.6%	3.3%		
10	*P.kishidana*-BC ZSM Lep 07934	8.1%	7.9%	8.4%	8.7%	8.7%	5.6%	5.6%	2.9%	2.0%	

##### ﻿Key to *Prochasma* species, based on characters of male genitalia

**Table:** 

1	Apical region of valva with excavation or incision on dorsal side	**2**
–	Apical region of valva straight on dorsal side	**3**
2	Valva narrow, apex with a deep excavation dorsally, a brush of elongated, modified setae present near excavation; cucullus indistinct	***P.diaoluoensis* sp. nov. (Hainan)**
–	Valva and costa broad, dorsally near apex with deep incision between both	***P.albimonilis* (Myanmar, Laos, Vietnam)**
3	Cornutus on vesica narrow, stick-like	**4**
–	Cornutus on vesica not stick-like	**5**
4	Cornutus long, about half the length of aedeagus; dentate process on ventral margin of valva not prominent	***P.mimica* (India, Assam)**
–	Cornutus shorter than one-third of aedeagus in length; dentate process on ventral margin of valva prominent	***P.kishidana* (Peninsular Malaysia, Borneo, Sumatra)**
5	Apex of uncus stout and short; dentate process on ventral margin of valva rather short	**6**
–	Apex of uncus slightly elongated; dentate process on ventral margin of valva slightly longer	**7**
6	Valva narrow; tapering part of cornutus long; dentate process on ventral margin of valva conspicuous	***P.squalida* (Taiwan)**
–	Valva broad; tapering part of cornutus shorter; dentate process on ventral margin of valva hardly visible	***P.parasqualida* (Vietnam, Laos, Thailand)**
7	Cornutus large, long, base not bulbous, tapering part less than one-third the length of cornutus, with short, acute tip	***P.dentilinea* (India, Nepal, Myanmar, Thailand, Laos, Vietnam)**
–	Cornutus smaller, base bulbous, tapering part nearly half the length of cornutus	***P.sasakiana* (Borneo)**

##### ﻿Checklist of the *Prochasma* species

### ﻿Genus Prochasma

Taxon classificationAnimaliaLepidopteraGeometridae

Warren

6B852B82-8788-5940-9688-99D63DC59AFD

Prochasma Warren, 1897, Novit. zool. 4: 81. Type species: Prochasmamimica Warren, 1897.

### ﻿ Prochasma mimica

Taxon classificationAnimaliaLepidopteraGeometridae

Warren

088B45AE-3868-5343-9145-F7D398CB29D5

Prochasma mimica Warren, 1897, Novit. zool. 4: 81. Type-locality: Khasi Hills, India.
Boarmia flavisecta Hampson, 1898, unnecessary replacement name for Prochasma “*minima*” Hampson, nec Warren.

#### Distribution.

India.

#### Remarks.

Only three specimens are known so far (collection of Natural History Museum, London). Sato (2019) figures a male and a female syntype (figs 1, 2) and male and female genitalia of syntypes (figs 27, 28).

### ﻿ Prochasma albimonilis

Taxon classificationAnimaliaLepidopteraGeometridae

Prout

FF47909F-FE00-5B44-B754-D2BBC39B49A7

Prochasma albimonilis Prout, 1927, J. Bombay nat. Hist. Soc. 31 (4): 943. Type-locality: Htawgaw, Burma; Sato 2019, Tinea 25 (Suppl. 1): 147; Sato 2020, Tinea 25 (Suppl. 2): 84, pl. 29, figs 25, 26.

#### Distribution.

Myanmar, Laos, Vietnam.

#### Remarks.

Sato (2019) figures the male holotype from NE Myanmar (fig. 5), a male and a female from Vietnam (figs 22, 23) and their genitalia (figs 36, 40).

### ﻿ Prochasma dentilinea

Taxon classificationAnimaliaLepidopteraGeometridae

(Warren)

7C48E6F3-9EFB-541B-A1FF-912D6084394A

Psilalcis dentilinea Warren, 1893, Proc. zool. Soc. Lond. (2): 431. Type-locality: Naga Hills, Sikkim, India.
Boarmia dentilinea : Hampson 1895, Fauna Br. India (Moths), 3: 277.
Prochasma dentilinea : Prout 1926, Sarawak Mus. J. 3 (2): 207; Prout 1932, J. fed. Malay. St. Mus. 17: 106; Sato 2019, Tinea 25 (Suppl. 1): 139.

#### Distribution.

India, Nepal, Myanmar, Thailand, Laos, Vietnam, SW. China (Han H. Beijing, pers. comm.).

#### Remarks.

Sato (2019) figures a male syntype from Naga Hills, E. India (fig. 3) and three males and one female from Nepal, Vietnam and Myanmar (figs 7–10), genitalia of male syntype (fig. 29), male and female genitalia from Myanmar (figs 30, 37).

### ﻿ Prochasma kishidana

Taxon classificationAnimaliaLepidopteraGeometridae

Sato

6252CF77-AC05-5BE1-AC93-969A2C530637

Prochasma kishidana Sato, 2019, Tinea 25 (Suppl. 1): 138–149, figs 11–13 (adults of holotype and paratypes, males and female), 32, 38 (genitalia of male and female). Type-locality: Holzweg, Prapat, Sumatera Utara, N Sumatra, Indonesia.

#### Distribution.

Peninsular Malaysia, Borneo (Brunei, Sarawak), Sumatra.

#### Remarks.

Specimens from Borneo have earlier been treated as *P.dentilinea* Warren (Holloway [1994], fig. 574, male genitalia, pl. 17, fig. 36, male adult). Both clearly belong to *P.kishidana*. The female genitalia (fig. 578) is different to those figured by Sato (2019, figs 38, 39) for *kishidana* and *sasakiana*, and may belong to a third, still unknown Bornean species, or the difference may be due to geographical variation, as Sato’s figure represents a female from Sumatra.

### ﻿ Prochasma parasqualida

Taxon classificationAnimaliaLepidopteraGeometridae

Sato

89D21872-CD43-54E5-9376-3563CB90E5E6

Prochasma parasqualida Sato, 2023, Tinea 26 (4): 379–385, figs 9–12 (adults of male holotype and female paratype), 16, 19 (male and female genitalia of syntypes). Type-locality: Ban Kalo, Phou Khoun, Luang Prabang, Laos.
Prochasma squalida : Sato 2019, Tinea 25 (Suppl. 1): 138–149, figs 19–21, 35, 42; Sato 2020, Tinea 25 (Suppl. 2): 84, pl. 29, fig. 24.

#### Distribution.

Vietnam, Laos, Thailand.

#### Remarks.

Considered as conspecific with *P.squalida* in Sato (2019, 2020).

### ﻿ Prochasma sasakiana

Taxon classificationAnimaliaLepidopteraGeometridae

Sato

D7B3C6D5-B16B-5740-92A1-B9A9CD168AE6

Prochasma sasakiana Sato, 2019, Tinea 25 (Suppl. 1): 138–149. figs 14–16 (adults of male holotype and female paratypes), 33, 39 (genitalia of male and female). Type-locality: Trus Madi Mt, Sabah, Borneo.

#### Distribution.

Borneo (Sabah).

#### Remarks.

Occurring together with *P.kishidana* on Borneo.

### ﻿ Prochasma squalida

Taxon classificationAnimaliaLepidopteraGeometridae

(Wileman)

F7CF68B6-2989-565F-992F-BD909987C84A

Boarmia squalida Wileman, 1915, Entomologist 48: 282. Type-locality: “Arizan, Formosa” (Alishan, Taiwan, China).
Prochasma dentilinea : Prout 1927, J. Bombay nat. Hist. Soc. 31 (4): 943; Inoue 1965, Spec. Bull. Lep. Soc. Japan 1: 34; Parsons et al. 1999, Geometrid Moths of the World, 782.
Prochasma squalida Sato, 2019, Tinea 25 (Suppl. 1): 138–149, (stat. rev.), figs 4 (male, holotype), 17, 18 (male, female, Taiwan), 34, 41 (male and female genitalia).

#### Distribution.

China (Taiwan).

#### Remarks.

This species had been sunk as a synonym of *Prochasmadentilinea* by Prout (1927), but was restored to a valid species by Sato (2019). Specimens from Vietnam, Laos and Thailand identified as conspecific with *P.squalida* in Sato (2019, 2020) were separated and treated as a new species, *P.parasqualida* in Sato (2023).

## Supplementary Material

XML Treatment for Prochasma

XML Treatment for Prochasma diaoluoensis

XML Treatment for Prochasma

XML Treatment for Prochasma mimica

XML Treatment for Prochasma albimonilis

XML Treatment for Prochasma dentilinea

XML Treatment for Prochasma kishidana

XML Treatment for Prochasma parasqualida

XML Treatment for Prochasma sasakiana

XML Treatment for Prochasma squalida

## ﻿Additional information

### Author ORCIDs

Bo Liu https://orcid.org/0009-0008-7003-4659

Dieter Stüning https://orcid.org/0000-0002-1748-4510

### Data availability

All of the data that support the findings of this study are available in the main text.
